# “Wire Syndrome” Following Bonded Orthodontic Retainers: A Systematic Review of the Literature

**DOI:** 10.3390/healthcare10020379

**Published:** 2022-02-17

**Authors:** Carole Charavet, France Vives, Sofia Aroca, Sophie-Myriam Dridi

**Affiliations:** 1 Université Côte d’Azur, Faculté de Chirurgie Dentaire, Département d’Orthodontie, 06000 Nice, France; f.vives@hotmail.fr; 2 Centre Hospitalier Universitaire (CHU) de Nice, Pôle Odontologie, UEC Orthodontie, 06000 Nice, France; 3 Laboratoire MICORALIS UPR 7354, Université Côte d’Azur, 06000 Nice, France; Sophie-Myriam.DRIDI@univ-cotedazur.fr; 4 Pratique Privée, 75116 Paris, France; sofiaaroca@me.com; 5 University of Bern, Department of Periodontology, 3012 Bern, Switzerland; 6 Université Côte d’Azur, Faculté de Chirurgie Dentaire, Département de Parodontologie, 06000 Nice, France; 7 Centre Hospitalier Universitaire (CHU) de Nice, Pôle Odontologie, UEC Parodontologie, 06000 Nice, France

**Keywords:** retainer, bonded retainer, fixed retainer, orthodontic retainer, wire syndrome, unexpected movement, unwanted movement, wire retainer

## Abstract

(1) Background and objective: Tooth movements described as unexplained, aberrant, unexpected, unwanted, or undesirable can occur in the presence of an intact orthodontic retention wire, without detachment or fracture. This iatrogenic phenomenon, known little or not by many practitioners, responsible for significant dental and periodontal complications, both functional and aesthetic, is called “Wire Syndrome” (WS). It is therefore considered an undesirable event of bonded orthodontic retainers, which must be differentiated from an orthodontic relapse. The objective was to perform, for the first time, a systematic review of the literature in order to define the prevalence of WS and to study its associated clinical characteristics. (2) Methods: A systematic review of the literature was performed following the guidelines of Preferred Reporting Items for Systematic Reviews and Meta-Analyses (PRISMA) and recommendations using an electronic search strategy on four databases complemented by a manual search. All the prospective and retrospective clinical studies, including case reports and series, written in English or French, clearly mentioning the description, detection, or management of WS were included. Three independent blinding review authors were involved in study selection, data extraction, and bias assessment using the Mixed Methods Appraisal Tool (MMAT). (3) Results: Of 1891 results, 20 articles published between 2007 and 2021 fulfilled the inclusion criteria, with a globally high risk of bias since 16 articles were case report/series. The analysis of each article allowed the highlighting of WS through 13 categories, as follows: prevalence, apparition delay, patient characteristics, arch and tooth involved, families of movements, dental and periodontal consequences, type of wire, risk factors, etiologies, treatment, and preventive approach. (4) Conclusion: This systematic review of the literature elaborated a synthesis on WS, allowing general practitioners, periodontists, and orthodontists to understand this adverse event, to facilitate the diagnostic approach, and to underline preventive measures against WS. This review was registered in the International Prospective Register of Systematic Reviews (PROSPERO; number CRD42021269297).

## 1. Introduction

The long-term follow-up of orthodontic bonded retainers remains a challenge for orthodontists, but also for periodontists, as well as general practitioners. Whereas fixed retainer placement is a common procedure after orthodontic treatment, complications, which can be severe, can happen. Indeed, in 2007, Katsaros et al. [1] were the first to describe this problem, which occurs when the orthodontic retainer is always bonded to the anterior teeth, inducing serious complications on these teeth under the name “unexpected complications of bonded mandibular lingual retainers”. This phenomenon was thereafter described by some authors under different names, such as “severe complication of a bonded mandibular lingual retainer” [2] in 2012, “Syndrome du Fil” [3] in 2015, “inadvertent tooth movement with fixed lingual retainers” [4] in 2016, or “extreme complication of a fixed lingual mandibular lingual retainer” [5] in 2021.

The synthesis of clinical experience evoked by the authors cited above allows us to define and characterize Wire Syndrome (WS) as follows: Fixed orthodontic retainers can provoke aberrant, unexpected, unwanted, or unexplained tooth movement on teeth still bonded by a fixed retainer placed after orthodontic treatment, which could induce progressively iatrogenic dental and periodontal complications, functional and/or aesthetic, ranging from minor teeth displacement to teeth expulsion from the bone with loss of vitality. In the presence of severe WS, the retainer may become detached or fractured. WS is not a classic orthodontic relapse, and the position of the teeth does not correspond to any previous situation.

However, neither general practitioners nor dental specialists, such as orthodontists and periodontists, are aware of the Wire Syndrome phenomenon. Concerning general practitioners, a lack of knowledge has been detected. A survey in eastern France showed that only 18.6% of general dentists were aware of the risks of adverse tooth movement associated with unintentionally active fixed retainers [6]; these results are globally in agreement with a Swiss survey by Habegger et al. [7]. However, general practitioners are seeing an increasing number of patients with a bonded retainer, estimated at 2–10 patients per week [7]. Concerning orthodontists, in a survey conducted by Padmos et al. [8] in New Zealand, one in eight was not familiar with this problem, and one in five had never seen any such cases. Padmos et al. [8] therefore concluded that it is necessary for all dental professionals worldwide to become more knowledgeable about this phenomenon, to be able to recognize associated cases, and also to prevent the worsening of complications. 

Therefore, the aim of this study was to perform the first systematic review of the literature on Wire Syndrome (WS) in order to define the prevalence, to study its associated clinical characteristics, and, specifically, to facilitate the diagnostic approach of practitioners and to underline preventive and curative measures against WS.

## 2. Methods

### 2.1. Protocol Registration

A systematic review of the literature (SRL) was performed, following as closely as possible the guidelines of Preferred Reporting Items for Systematic Reviews and Meta-Analyses (PRISMA) and recommendations (reference). The protocol was registered in the International Prospective Register of Systematic Review (PROSPERO) (CRD42021269297).

### 2.2. Article Identification

#### 2.2.1. PICOs Question and Eligibility Criteria

According to the question formulated using the “Population, Intervention, Comparison, Outcome and Study Designs (PICOs) model”:

– Participants (P): Patients with “Wire Syndrome” (WS), i.e., dental movements described as aberrant, unexpected, unexplained, unwanted, or excessive;
– Interventions (I): Fixed orthodontic retainer bonded at the maxilla and/or mandible after orthodontic treatment;
– Comparisons (C): Patients not affected by “Wire Syndrome” (only for studies including a control group);
– Outcomes (O): Define the prevalence of “Wire Syndrome” and the characteristics associated with it.
– Study designs (S): All prospective and retrospective clinical studies, including case reports or series, written in English or French, clearly reporting the description, detection, or management of “Wire Syndrome” or tooth movement described as aberrant, unexpected, unwanted, or unexplained in the presence of a bonded fixed retainer placed after orthodontic treatment were included, regardless of the length of the follow-up. In vitro studies, narrative reviews, author opinions, editorials, or commentaries were excluded.

#### 2.2.2. Search Strategy

❖ Electronic search

A search strategy, tailored to each database, combining keywords, Medical Subject Headings (MeSH) terms, and Boolean operators was performed without date restriction.

The electronic search was conducted on four different databases on 1 September 2021 (Table 1).

❖ Manual search

To complement the electronic searches, a manual search was conducted:

– From the bibliography of articles selected by the electronic search;
– From the search engine of a selection of orthodontic and dental journals:
  ○ American Journal of Orthodontics and Dentofacial Orthopedics;
  ○ European Journal of Orthodontics;
  ○ Journal of Orthodontics;
  ○ Journal of Clinical Orthodontics;
  ○ Orthodontic & Craniofacial Research;
  ○ The Angle Orthodontist;
  ○ Revue d’Orthopédie Dento-Faciale;
  ○ L’information Dentaire.

### 2.3. Article Selection

#### 2.3.1. Electronic Search

This research was conducted using the reference management software Zotero version 5.0.96.2 (https://www.zotero.org accessed on 1 September 2021). Two authors carried out the entire procedure independently. In the case of disagreement, a third author was interviewed, and a mutual discussion was conducted to reach a consensus.

After avoiding duplicates, the inclusion of articles was carried out in three steps:

– Reading of titles;
– Reading of abstracts;
– Reading of the full text.

For articles with no available abstracts, full-text articles were read for eligibility assessment.

#### 2.3.2. Manual Search

The same selection procedure applied to the electronic research was carried out.

### 2.4. Data Extraction

For the articles selected in this SRL, tables were made to synthesize the important data of the articles by the same two operators independently, as follows: author names, year of publication and journal, aim, study design, population, and results summary.

Because the included studies had heterogeneous methods, methodological analyses, and results, it was not possible to perform a meta-analysis. Therefore, the analysis of the articles was qualitative and descriptive.

### 2.5. Risk of Bias Analysis

Given the heterogeneity of the studies selected for this SRL, a tool that can evaluate the methodological quality of different types of studies within the same SRL was employed: The “Mixed Methods Appraisal Tool” (MMAT) [9] (http://mixedmethodsappraisaltoolpublic.pbworks.com accessed on 1 September 2021).

## 3. Results

### 3.1. Article Selection

#### 3.1.1. Electronic Search

The electronic search conducted on 1 September 2021 identified 1891 references. After eliminating duplicates, 1270 references were analyzed. Based on the reading of the title, 211 titles were retained. Then, a reading of the abstract was conducted, retaining 50 articles for a full reading of the text. After a complete reading of these 50 articles, 15 articles were finally retained.

#### 3.1.2. Manual Search

The manual search identified 13 additional articles. After applying the selection procedure on these 13 articles, five articles were retained in the present study.

**Figure 1:**
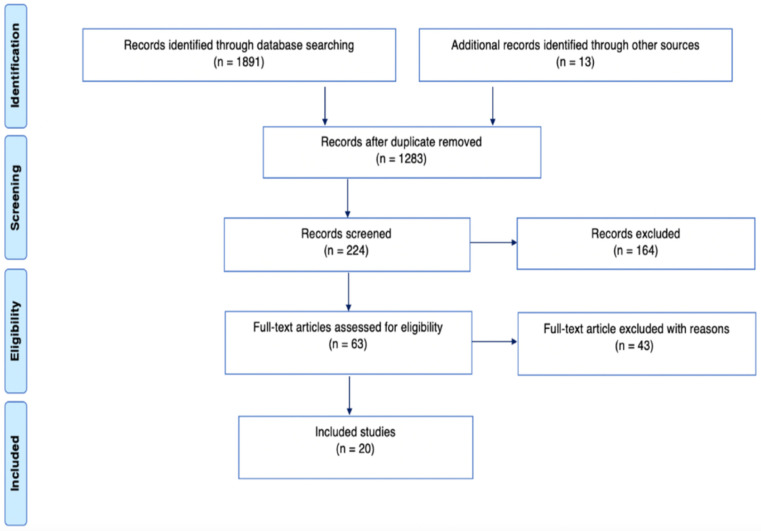
Systematic search and selection strategy. PRISMA flow diagram.

In total, the applied search strategy resulted in the selection of 20 articles in this SRL (Figure 1).

### 3.2. Studies’ Characteristics

Out of the 20 articles selected in our SRL, the study designs were heterogeneous, with one randomized controlled study [10] and a large majority of case series and case reports. 

Additionally, the included studies were conducted worldwide, and the year of publication varied from 2007 to 2021.

Several different names were attributed to “Wire Syndrome” (Table 2). More specifically, the first article describing “unexpected movements” was by Katsaros et al. [1] in 2007. The French term “Syndrome du Fil” was introduced thereafter by Roussarie et al. [3] in 2015. 

The terms “X effect” and “Twist effect” were first used by Kucera et al. [11] in 2016. 

Finally, no conflict of interest in any studies were declared.

**Table 2:** History of the different definitions/descriptions of Wire Syndrome (WS).

Authors	Publication Date	Wire Syndrome Definition
Katsaros et al. [1]	2007	Unexpected complications of bonded mandibular lingual retainers
Abudiak et al. [12]	2011	A complication with orthodontic fixed retainers
Renkema et al. [13]	2011	Unexpected posttreatment complications
Alessandri Bonetti et al. [14]	2012	Isolated-type recession defects with an abnormal buccolingual inclination
Pazera et al. [2]	2012	Severe complication of a bonded mandibular lingual retainer
Farret et al. [15]	2015	Extreme labial movement of the root
Roussarie et al. [3]	2015	Syndrome du fil
Kučera et al. [11,16]	2016	Unexpected complications/X effect, Twist effect, and non-specific complications
Laursen et al. [17]	2016	Complications after unintentional tooth displacement by active bonded retainers
Shaughnessy et al. [4]	2016	Inadvertent tooth movement with fixed lingual retainers
Wolf et al. [18]	2016	Undesired tooth movement
Egli et al. [10]	2017	Unexpected posttreatment changes
Jacobs et al. [19]	2017	Single tooth torque problems
Beitlitum et al. [20]	2020	Unwanted effects such as inadvertent tooth movement and torque changes
Kim et al. [21]	2020	Unexpected tooth movements
Klaus et al. [22]	2020	Unwanted tooth movements
Knaup et al. [23]	2021	Side effects of twistflex retainers
Singh et al. [5]	2021	Extreme complication of a fixed mandibular lingual retainer

### 3.3. Bias Results

The MMAT score was relatively high for each included study, with a mean of 73 ± 18.2%.

### 3.4. Studies’ Results

A description of each included study is provided in Table 3. 

Analysis of the results allowed the synthesis of said results around 13 categories. Note that not all articles presented information in all categories.

Prevalence: The prevalence varied from 1.1% to 43.0%. The lowest prevalence estimate of 1.1% was found in a retrospective study by Kučera et al. [11], which had the largest sample size, with 3500 patients included. The highest prevalence was found in the study of Wolf et al. [18], which included only 30 patients, with a prevalence of 13% and 30% in severe and moderate WS, respectively.

Apparition delay: Kucera et al. [11] showed that WS appeared in a mean interval of 4 ± 2.8 years. The apparition delay found in the included case reports and series was mostly (80%) within this range, except for five publications that were above this range [3,4,15,17]. The shortest apparition delay reported was one year in the study of Katsaros et al. [1], and the longest time reported was 21 years after placement of a bonded retainer in the case report of Farret et al. [15].

Patient characteristics: Gender: The overall publications included 40 men and 81 women. *Age:* The youngest WS patient identified in this SRL was 13.5 years old [1], and the oldest was 56 years old [20]. The study by Kucera et al. [11] calculated the average age of its 38 WS patients and found it to be 20.7 ± 8.9 years. The ages of the patients included in the case reports and series mostly corresponded to this range, except for three [15,17,20], who were above this range. Parafunction: Only Alessandri Bonetti et al. [14] mentioned parafunctions, where, for two patients with WS, onychophagia was demonstrated by questioning and exobuccal examination (nail deformation).

Arch and tooth involved: WS was found in 72 cases in the maxilla versus 179 cases described in the mandible. Additionally, WS involved 39 maxillary incisors, 6 maxillary canines, 64 mandibular incisors, and 100 mandibular canines.

Families of movements: Although WS shows significant interindividual variation, the movements can be categorized into four groups (Table 4).

Dental consequences: WS is responsible for displacement of the affected teeth in all spatial dimensions with a large range of variations. These displacements can be slight, according to the study by Klaus et al. [22], which mentioned a median magnitude of unwanted movement from 0 to 0.66 mm. However, extreme movement of the tooth can cause rupture of the vascular–nervous bundle and loss of vitality of the tooth [14,15] associated with the exposure of the root until the apex [5].

Periodontal consequences: Tooth WS displacements can contribute to vestibular or lingual gingival recessions, dehiscences, or fenestrations apparition, responsible for aesthetic repercussions and/or exposure of the root, leading to discomfort or pain, particularly due to hypersensitivity [20]. In severe cases, displacement of the root out of its socket may result in resorption of the entire vestibular or lingual wall, depending on the direction of tooth movement [15]. The root (and its apex) may be projected out of the bone [2].

Type of retention wire: Several different types of wires are involved in the occurrence and development of WS, as follows:

❖ Flat, braided chains (Ortho FlexTech).
❖ Single-stranded, round wire bonded on the canines only; diameter: 0.036 inch.
❖ Round, twisted, stainless steel wire:
  - Unknows strands with diameter: 0.0175 inch; 0.0215 inch; 0.0195 inch;
  - Three strands with a diameter of 0.0155, 0.0195, or 0.0195 inches (heat treatment);
  - Five strands with a diameter of 0.0215 inched (gold-plated);
  - Six strands with a diameter of 0.0175 inches.
❖ Round, coaxial, stainless steel wire:
  - Six strands with a diameter of 0.018 inches.

Most cases of WS are seen in the presence of round, twisted, stainless steel wires [1,2,3,4,5,10,11,12,13,14,16,17,18,21,23], although flat, braided chains are also concerned [3], as well as single-stranded, round wires bonded only on the canines [5] and round, coaxial, stainless steel wires [22]. 

Risk Factors: Predisposing factors of WS were investigated in three studies [11,18,22]:

❖ Patient-related factors: Different parameters were found to be significant in WS patients, such as lower facial level increase (*p* < 0.0001) [11], vestibulo-position of mandibular incisors before orthodontic treatment (*p* = 0.029) [11], and presence of dysfunctions/parafunctions (*p* = 0.049) [22]. However, Klaus et al. [22] did not find any significant difference in WS patients regarding mandibular plane angle or initial vestibulo-version of the incisors.
❖ Orthodontic treatment-related factors: Different parameters were found to be significant in WS patients, such as debonded at a young age (*p* = 0.03) [11], canine expansion and overjet reduction during treatment [18], and absence of inter-incisal contact at the end of treatment (*p* < 0.01) [22]. In contrast, Klaus et al. [22] found no significant difference in WS patients regarding expansion of the inter-canine distance and reduction in overjet. Kucera et al. [11] showed no significant difference regarding treatment duration (*p* = 0.270), inter-canine distance (*p* = 0.065), or change in incisor inclination (*p* = 0.151).
❖ Wire-related factors: No significant differences were found in patients with WS regarding debonded wire (*p* ≤ 0.05) [22], (*p* = 0.562) [11] and type of wire (*p* = 1.000) [11].

Etiologies: Different etiological hypotheses were mentioned in the included studies, which can be grouped into three categories, as follows (Table 5): practitioner-, wire-, and patient-related etiologies.

Treatment: Treatment depends on the severity of WS. It should be noted that in the three cases where treatment was not performed [14,21], clinical aggravation occurred.

❖ Mild severity: The most common treatment was retainer removal. Some authors [10,23] observed significant improvement up to spontaneous repositioning. Stripping was recommended by Roussarie et al. [3] to facilitate teeth repositioning and avoid relapse. The correction of parafunctions was also recommended [14]. For two research teams [3,5], an observation period of six months to one year was performed after wire removal.
❖ Moderate severity: The wire is also removed, but orthodontic retreatment is required to correct malposition and to properly reposition the root in the alveolar bone, in order to improve surgical conditions [2,3,12,14,15,19,20].
❖ Significant severity: Orthodontic retreatment combined with endodontic and/or periodontal treatment is indicated. Endodontic treatment is performed when the displacement of the tooth is so important that it has caused a rupture of the vascular–nervous bundle. Endodontic surgery may be associated if necessary [14,15]. In cases where periodontal surgery is indicated, the removal of retainers is beneficial [20]. In cases of extreme WS, dental avulsion is sometimes the only solution [5].

Recommendations: The authors of the included studies described some recommendations to avoid the development of WS (Table 6).

## 4. Discussion

To the best of our knowledge, the present publication is the first systematic review of the literature on the subject of Wire Syndrome (WS). After identifying 1891 articles, 20 articles were selected and analyzed, with a globally high risk of bias. Given the limited number of existing publications on WS and the relevant information found in the case reports and case series, these types of study designs were included. 

The description of WS is recent; the first publication on WS appeared in 2007 [1], followed by an increase in publications, and the distinction between classic relapse and WS is also a very new concept. Since the introduction of fixed bonded retainers in the 1970s by Zachrisson, fixed retainers have been progressively preferred to removable thermoplastic application by patients and practitioners [22,25], and, parallelly, there has been an increasing number of orthodontic treatments. The prevalence of WS is therefore likely to rise and also to attract more research interest. Concerning specifically the prevalence of WS, six studies estimated the prevalence of adverse movements associated with bonded retainers, in which the prevalence varied from 1% (3500 patients studied [11]) to 43% (30 patients studied [18]). This wide range can be explained by the differences in sample size between these two monocentric studies. Moreover, the protocols (patient selection, practitioners, type of wire, bonded protocol, etc.) were very different between the studies. 

Regarding the delay in apparition, WS appeared between 1 and 21 years after the placement of a retainer in the included studies. The majority of cases of WS appeared within the first five years after the placement of the retainer. This time interval should be considered with caution for several reasons. First of all, it is difficult to date the apparition of WS with precision, as retainer visits are intermittent. Moreover, it is difficult to detect early WS, and the patient often consults when complications are already severe [20]. Concerning the characteristics of the patients, all ages, particularly young people under 30 years of age, are affected by WS. Twice as many women as men seem to be affected. Orthodontic treatment is usually performed in adolescence, and WS occurs also a few years after the end of orthodontic treatment. In addition, one study showed the presence of onychophagia in both patients with WS [14]. This parafunction could therefore increase the risk of developing WS in our patients. Additionally, concerning the arch and tooth concerned, WS was first described in the mandible in 2007 by Katsaros et al. [1], although the first case described in the maxillary arch was in 2011 [12]. Moreover, although in the study by Klaus et al. [22] maxillary teeth are more affected by WS than mandibular teeth (20.9% versus 14%), most WS cases occur in the mandible. Indeed, more fixed retainers are placed in the mandible than in the maxilla, where thermoplastic retainers are preferred [8,26]. Placement of a maxillary bonded retainer requires composite plots that can create interference/occlusal trauma with the mandibular arch, resulting in more failures [27]. In addition, when a maxillary bonded retainer is indicated, wires are most often placed from lateral incisor to the lateral incisor than from canine to canine to avoid the previously mentioned difficulties [28], so WS on maxillary canines is rare. However, it should also be noted that WS in the maxilla is more quickly detected than WS in the mandible because it affects the patient’s smile, which leads to early consultation [3]. Finally, the teeth most often affected by WS are the mandibular canines. It would seem that WS preferentially affects the “terminal” teeth that are always contained by the fixed bonded retainer.

In the presence of WS, the bonded retainer is intact in most cases but, in severe cases, the wire may become partially debonded or fractured due to important dental movement. In any situation, various and different clinical dental and periodontal signs can be found. The detection of one sign related to WS must immediately alert the practitioner to the possible presence of WS in order to stop the iatrogenic evolutive process and to start adapted therapy. Indeed, this syndrome is progressive and starts with minor dental and periodontal consequences until the loss of vitality and/or tooth expulsion. In addition, when treatment is not carried out [14,21], clinical worsening can occur [2,24]. Movements due to WS do not correspond to a relapse or a physiological process: The situation of the teeth does not correspond to their position before orthodontic treatment, nor to their position at the time of debonding, as underlined by Kastaros et al. [1]. WS can therefore be qualified as a new malposition observed after placement of a fixed bonded retainer following orthodontic treatment [18,22], although no value or threshold has been scientifically determined [22]. Thus, a severe WS is easily identified because the clinical signs are more marked, contrary to an early WS, whose clinical signs are mild; the identification of complex cases is easy, while early detection remains difficult. In the case of mild movements, differentiation of movements related to a classic relapse from WS also remains arduous. Therefore, the challenge for orthodontists during follow-up visits is to detect incipient WS. Additionally, general practitioners, as well as periodontists, also have an important role to play in the early detection of WS. Finally, the patient must also be actively involved in monitoring [1,13]. Patients should be alerted to the need for maintenance and the plausible occurrence of adverse effects related to the presence of the retainer wire. 

With regard to WS prevention, the most important preventive measure is the use of a bonded passive retainer. The use of a dental model to perfectly fit the retainer to the teeth before placement is recommended, as well as an indirect bonding protocol. Furthermore, special care should also be taken when the fixed retainer needs to be repaired. When a composite comes loose, small tooth movements may have already occurred and the wire is no longer a perfect fit. In this case, using an instrument to “push” the wire to better fit the teeth results in an active wire that could potentially be responsible for subsequent WS. Therefore, a new passive wire should be bonded rather than repaired. Another strategy for preventing WS is double retention, which combines a fixed bonded retainer with a removable thermoplastic retainer [4,5,8,18]. Finally, the number of WS cases increases during the first five years according to Kucera et al. [11], suggesting that monitoring in the orthodontic office should be preferred, at least during this interval. Treatment depends on the severity of the case, from non-invasive treatment, through a multidisciplinary endodontic/periodontal approach, to extraction of the involved tooth in extreme cases. In the case of early WS, the most important reflex is to remove the fixed retainer to immediately stop the iatrogenic WS process; spontaneous correction of tooth malposition may occur [10,23].

Although there are explanatory hypotheses that could justify the risk factors mentioned by the authors, not all studies agreed. Indeed, WS seems to be due to a combination of different and multifactorial etiologies. In addition, it appears that the delay in apparition varies with etiology [29]. Early WS could probably be explained by an error in wire adaptation (lack of passivity) or bonding [29]. When WS appears several years after orthodontic placement, wire-related etiologies are preferred. Regarding dysfunction, some authors hypothesized that oro-vestibular forces exerted by the tongue could cause undesirable movement [2,22,29], although Shaugnessy et al. excluded the role of the tongue because its pressure would be less than that required to deform the wire [4]. In addition, the risk of wire deformation also increases with time due to the progressive wear of the composite, which results in a larger section of the wire being exposed to deformation [29]. Additionally, a change or instability in the mechanical properties of the wires, whether inborn or acquired, could be involved in WS. A fixed retainer could become unintentionally active. Moreover, a break in adhesion at the wire–composite interface can cause a “pivot effect”, resulting in torsion of the teeth around the wire, which then acts as a center of rotation [8,15,29]. The tooth pivots around the wire, which could explain the unwanted torque of the teeth involved in WS. After observing the rotational movements of the six anterior teeth, Wolf et al. [18] hypothesized that the fixed wire causes forces that rotate the entire block of interconnected anterior teeth stiffened by the wire in the vestibular direction on one side and lingual on the other due to physiological transverse constriction. 

The type of wire seems to have an influence on the occurrence of WS. In the study of Padmos et al. [25], the mechanical properties of round multistrand wires were incriminated. Indeed, most of the included studies in this systematic review described WS as being associated with round, multistrand twisted wires [1,2,3,4,5,10,11,12,13,14,16,17,18,21,23]. Engeler et al. [30] assured that all documented adverse movements are present only with multistrand wires, whereas Roussarie et al. [24] showed that no wire is immune to WS. While some authors expressed a preference for the type of wire to be used to avoid the development of WS, no consensus on wire selection could be advanced. In addition, the diversity of materials and bonding protocols make it difficult to draw any conclusions. Recently, Gelin et al. [31] investigated the effect of rectangular, 0.014 × 0.014 inch, memory shape-customized CAD/CAM nitinol retainers (Memotain ^TM^; CA Digital GmbH, Mettmann, Germany) versus round, 0.0175 inch (in), six-stranded, twisted, stainless steel wire retainers (Supra-FlexTM; RMO Europe, Illkirch-Graffenstaden, France) and showed no significant difference between these two types of retainer after one year of placement. To reach a consensus on the preferred type of wire to use, randomized controlled studies must be conducted on a large sample size and over long observation periods.

Finally, some additional points should be made regarding this systematic review of the literature. First, only one study designed as a randomized controlled trial was included for analysis, and the risk of bias was considered high, as the majority of included studies were designed as case series/reports. Second, there was a lack of information in the included studies. For example, few data were provided on patient characteristics prior to orthodontic treatment (e.g., baseline crowding and cephalometric measurement), orthodontic biomechanics employed to treat the patient (e.g., elastics employed and brackets prescribed), bonding and retainer placement protocol, history of retainer failures (e.g., number of breakages, detachment, or re-bonding), etc. Furthermore, the periodontal conditions, such as type of phenotype [32], traction of labial frenum, or oral hygiene quality, were not investigated in each study. However, these parameters may have an influence on the development of WS, probably from a multifactorial origin. Moreover, the selected studies have extreme heterogeneity in terms of the variables compared and the outcomes measured. Concomitantly, the enormous heterogeneity of the included studies made it impossible to perform a meta-analysis. All of this represents a limitation that is important to consider. 

## 5. Conclusions

This first review of the literature on Wire Syndrome (WS) included 20 articles published between 2007 and 2021, with a majority of case report/series leading to a globally high risk of bias. However, the analysis of the overall article provided an understanding of this adverse event associated with fixed orthodontic retainers, emphasized the importance of an early diagnosis, and highlighted preventive measures against WS for dental professionals worldwide, including general practitioners (GP), periodontists, and orthodontists. Indeed, the WS problem must involve all the dental health professions, including the general practitioners who will be able to refer, if necessary, the patient to a specialist practitioner; the continuity of the collaboration and the “ortho–paro–gp” link will then be prolonged during the therapeutic time, thus guaranteeing optimal patient care. Further studies are needed to improve the knowledge about fixed orthodontic retainers, based on a large, well-documented sample and conducted over a very long observation period.

## Figures and Tables

**Table 1:** Search strategy, according to each database.

Data Base	Search Strategy
PubMed (MEDLINE)	(orthodontic retainer) AND (((complication OR movement OR post treatment changes OR version) AND (unexpected OR unwanted OR inadvertent)) OR (“orthodontic retainers/adverse effects” (MeSH Terms)) OR (Side effect) OR (Relapse) OR (Torque) OR (twist effect) OR (Active)
The Cochrane Library	(orthodontic retainer) AND (((complication OR movement OR post treatment changes OR version) AND (unexpected OR unwanted OR inadvertent)) OR (adverse event) OR (Side effect) OR (Relapse) OR (Torque) OR (twist effect) OR (Active)
Embase	(orthodontic retainer) AND (((complication OR movement OR post treatment changes OR version) AND (unexpected OR unwanted OR inadvertent)) OR (adverse event) OR (Side effect) OR (Relapse) OR (Torque) OR (twist effect) OR (Active)
Scopus	((orthodontic AND retainer) AND (((complication OR movement OR “post treatment changes” OR version) AND (unexpected OR unwanted OR inadvertent)) OR (“adverse event”) OR (“Side effect”) OR (relapse) OR (torque) OR (“twist effect”) OR (active))

**Table 3:** Summary description of each included study. AJODO: American Journal of Orthodontics and Dentofacial Orthopedics. * If the article had several aims, only the one related to Wire Syndrome is mentioned.

Author (Year Published) Journal	Aim *	Study Design	Population	Main Results
**Katsaros et al. [1]** **(2007)** ***AJODO***	Demonstrate unexpected labiolingual changes in the mandibular anterior region associated with orthodontic bonded retainers.	Case series	Patients were screened for unexpected posttreatment changes in the mandibular anterior region during a three-year period for regular posttreatment follow-up.	21 patients presented unexpected movements, half of which required retreatment. **Prevalence:** 5%. **Arch involved:** Mandible. **Observed movements:** 18 patients had differences in torque between two adjacent mandibular incisors and 3 patients had significant buccal inclination and movement of one mandibular canine previously adapted on a working dental cast. **Retainer:** 0.0195 inch, three-strand, heat-treated twistflex wire bonded to the six mandibular anterior teeth.
**Abudiak et al. [12]** **(2011)** ***Orthodontic*** ***Update***	Describe a case of severe unwanted movement, the cause of which is believed to be the activation of a multistrand bonded retainer.	Case report	21-year-old patient completed a fixed appliance treatment five years ago and had a fixed maxilla and mandibular retainer bonded to all the teeth from canine to canine. She observed worsening displacement of teeth 12 and 13.	The patient presented unexpected movements. **Arch involved:** Maxilla. **Observed movements:** Open bite 13/43–44, height difference between the clinical crowns of 13 and adjacent teeth, excessive palatal root torque of 13, and excessive buccal root torque of 12 (apex palpable in the vestibule). **Retainer:** 0.0195-inch, three-strand, heat-treated twistflex wire bonded from canine to canine. **Apparition delay:** Two years. None of these unexpected movements were present at the end of the treatment and also did not correspond to the initial position of the teeth. **Treatment:** Wire removal; orthodontic retreatment; new 0.0195-inch, twisted, bonded passive retainer fabricated on a study model and placed using a jig.
**Renkeman et al. [13]** **(2011)** ***AJODO***	Long-term effectiveness of flexible spiral wire canine-to-canine lingual retainers in maintaining alignment of mandibular anterior teeth.	Case series	221 patients who received a flexible spiral wire canine-to-canine lingual retainer after active orthodontic treatment.	Of the 221 patients (75 boys and 146 girls), 6 patients presented unexpected movements, of which 3 required retreatment. **Prevalence:** 2.7%. **Arch involved:** Mandible. **Observed movements:** Five years after debonding, three patients had a torque difference between the two mandibular central incisors, two patients had an increased buccal inclination and movement of the mandibular left canine, and one patient had a torque difference between the two mandibular central incisors and increased buccal inclination and movement of the mandibular left canine. **Retainer:** 0.0195 inch, three-strand, heat-treated twist wire, bonded to the six mandibular anterior teeth.
**Alessandri Bonetti et al. [14]** **(2012)** ***AJODO***	Describe the diagnosis and management of isolated-type recession defects of complex etiology.	Case series	Two post-orthodontic patients (18 and 22 years old) presented gingival recession limited to one mandibular incisor associated with abnormal buccolingual inclination despite six-unit lingual bonded retainer.	Patients presented unexpected movements. **Observed movements:** First patient: Labial gingival recession and excessive buccal and lingual root inclination of 41 and 31, respectively. Second patient: Gingival recession and buccal dislocation of the root on 42. **Apparition delay:** First patient: Five years. Second patient: Four years. **Hypothetical etiology in both:** Onychophagia. **Retainer in both:** Round, twisted, stainless steel wire bonded to the six mandibular anterior teeth. **Treatment:** First patient: Onychophagia managing; removal retainer; orthodontic retreatment; periodontal surgery. Second patient: As the patient refused the treatment, a worsening was observed one year later that finally induced an acceptance of treatment, as follows: Endodontic treatment on 42 (vitality loss); orthodontic retreatment; periodontal surgery; a new multibraided, rectangular, stainless steel wire was bonded from canine to canine.
**Pazera et al. [2]** **(2012)** ***AJODO***	Present a severe complication of a lingual flexible spiral wire retainer.	Case report	20-year-old patient who previously underwent an orthodontic treatment. He came to the clinic with a fracture of his wire retainer four years after debonding.	The patient presented a serious complication. **Arch involved:** Mandible. **Observed movements:** Excessive buccal root torque (35°) on 43 with significant lingual inclination of crown and buccal gingival recession. CBCT revealed that the root and its apex were almost out of the buccal bone on its buccal side. Pulp vitality was preserved. **Apparition delay:** Four years. **Retainer:** Soft, twisted wire bonded to the six anterior teeth with a fracture between 42 and 43. **Treatment:** Orthodontic retreatment; new 0.0215 × 0.027 inch rounded steel wire bonded to the canines only; recession was still present but the patient refused periodontal treatment.
**Farret et al. [15]** **(2015)** ***AJODO***	Describe the case of a patient who underwent previous orthodontic treatment 21 years ago and had a fixed mandibular bonded retainer.	Case report	36-year-old patient who completed orthodontic treatment 21 years previously but had his mandibular bonded retainer partially debonded and broken for four years. He came to the clinic with pain and gingival recession on 32.	The patient presented unexpected movements. **Arch involved:** Mandible. **Observed movements:** Localized open bite 33/12–13, excessive buccal crown torque of 33, and extreme labial movement of the root of 32, but the vitality test was negative. **Apparition delay:** 21 years. **Retainer:** Wire (no detail provided) bonded to the six anterior teeth and fractured between 42 and 43. The mandibular left lateral incisor stayed bonded to the retainer and received the entire load of the incisors. **Treatment:** Orthodontic retreatment; endodontic treatment followed by apectomy of 32; a slight residual recession remained; a new 0.016 × 0.022 inch stainless steel mandibular fixed retainer was bonded to the mandible.
**Roussarie et al. [3]** **(2015)** ***Revue*** ***d’Orthopédie*** ***Dento-Faciale***	Describe Wire Syndrome associated with maxilla and mandibular bonded retainers.	Case series	60 patients presenting Wire Syndrome. Patient’s documentation came from Dr. Roussarie’s office and from colleagues.	60 patients presented Wire Syndrome (WS). **Arches involved:** Maxilla and mandible. **Observed movements:** Of the 40 cases observed in the mandible, 29 had a right canine with exaggerated vestibular crown torque, and 11 had a left canine with exaggerated lingual crown torque. Of the 20 cases observed in the maxilla, only 2 cases involved canines. **Retainer:** Three- or six-stranded twist round wires, flat braided chain, or 0.036 inch single-stranded wire bonded only on the canines.
**Kučera et al. [11]** **(2016)** ***AJODO***	Describe different types of unexpected complications associated with mandibular-fixed retainers, assessing their prevalence and possible etiological causes.	Retrospective cohort study	3500 consecutive patients (1423 men; 2077 women) who had a mandibular-fixed bonded retainer were screened for unexpected complications and then compared with a randomly selected control group of 105 patients (43 men; 62 women; 29.5 ± 9.7 years) without unexpected complications.	38 patients (20.7 ± 8.9 years) presented unexpected complications. **Prevalence:** 1.1%. **Arch involved:** Mandible **Observed movements:** 21 patients had an opposite inclination of the contralateral canines = twist effect (89.5% of the left canines were tipped buccally). 12 patients had a torque difference between two adjacent incisors = X effect. Five patients had nonspecific complications. **Apparition delay:** 4 ± 2.8 years post-treatment. The number of intercepted unexpected complications was highest in the first five years after debonding, and then it declined with time. **Retainer:** 0.0215 inch, gold-plated, five-stranded spiral wire OR a 0.0175 inch, six-stranded, co-axial wire bonded to the six anterior teeth. **Etiologies:** Patients in the “unexpected complications” group were, at pretreatment, with a higher mandibular plane angle (*p* < 0.0001), as well as the position of the mandibular incisors relative to the Point A-pogonion line (*p* = 0.029), but no difference was observed for intercanine distance (*p* = 0.065) or mandibular incisor inclination to the mandibular plane (*p* = 0.151) between the two groups. Patients in the “unexpected complications” group were also significantly younger at debonding (*p* = 0.03), but there was no significant difference in treatment time (*p* = 0.270), wire type (*p* = 1.000), or failure rate (*p* = 0.562) between the two groups.
**Kučera et al. [16]** **(2016)** ***Journal of Clinical Orthodontics***	Describe the interdisciplinary treatment of gingival recession secondary to an unexpected complication associated with a fixed mandibular retainer.	Case report	28-year-old patient completed two orthodontic treatments, in which 14/24 and a mandibular central incisor were extracted. The patient observed 43 gradually worsening.	The patient presented an unexpected complication. **Arch involved:** Mandible. **Observed movements:** An anterior open bite, a difference in height of the clinical crowns in the anterior sector, left canine and incisor inclined buccally, and right canine and incisor inclined lingually (twist effect). On 42, a gingival recession of 4 mm with exaggerated root prominence was observed. Finally, on the panoramic, the roots of 32 and 33 were tipped. **Retainer:** 0.0155 inch, three-stranded twisted wire, debonded of the lower left incisor. **Treatment:** Orthodontic retreatment; periodontal treatment; a new fixed retainer with a five-stranded, gold-plated wire of a 0.0215 inch diameter bonded on the six anterior teeth and extended to the first premolars.
**Laursen et al. [17]** **(2016)** ***Journal of Clinical Orthodontics***	Describe how to correct unwanted tooth movements with rational biomechanics.	Case series	Two patients (24 and 31 years old) completed orthodontic treatment and had a mandibular-fixed retainer.	The patients had unwanted movements. **Arch involved:** Mandible. **Observed movements:** First patient: 31 had an exaggerated labial root torque with a labial gingival recession. Second patient: 42 had exaggerated lingual root torque with a lingual gingival recession associated with lingual bone dehiscence. The tooth was still vital. **Apparition delay:** First patient: 10 years. Second patient: Five years. **Type of wire:** First patient: A flexible spiral wire. Second patient: A heat-treated, flexible spiral wire. **Treatment:** First patient: Retainer removal; orthodontic retreatment; periodontal treatment. Second patient: Retainer removal; orthodontic retreatment; periodontal treatment. In both cases, after retreatment, double retention: A three- (first patient) or six-stranded (second patient) spiral bonded wire associated with a vacuum-formed retainer for nighttime wear.
**Shaughnessy et al. [4]** **(2016)** ***AJODO***	Illustrate inadvertent tooth movement associated with fixed retainer, debate possible causes, make recommendations, and discuss orthodontic–periodontic management.	Case report with illustrated discussion	28-year-old patient that completed an orthodontic treatment 15 years prior and presented an intact fixed mandibular retainer. She had regular check-ups for the first year, but since then, no check-ups have been made.	The patient presented unwanted movements. **Arches involved:** Mandible. **Observed movements:** Gingival recessions, lingually on 42 and buccally on 41 with differential torque between 41 and 42. A difference in the height of the clinical crowns was observed on the anterior teeth. In the canines, the opposite inclination of 33 and 43 was noted. On the CBCT, bone fenestration was observed on 43 and 41 buccally and on 42 lingually. **Apparition delay:** 15 years. **Retainer:** 0.0195 inch twisted wire bonded to the six anterior teeth for the case report. **Treatment:** Retainer post-treatment; orthodontic retreatment; periodontal surgery; a removable retainer, according to the patient’s request.
**Wolf et al. [18]** **(2016)** ***Journal of Orofacial Orthopedics***	Analyzed post-treatment changes in the anterior mandibular region.	Case series	30 patients aged 24.52 ± 4.36 years completed orthodontic treatment (for at least one year of active treatment).	**Prevalence of severe adverse movement:** 13%, which required orthodontic retreatment. **Observed movements:** Superposition of each digitized and segmented tooth permitted to define the type of the movement to which each lower anterior tooth had been subjected, and in-depth analysis revealed that the canines underwent the most pronounced rotation and translation. **Retainer:** Dentaflex 0.45 mm, three-stranded twisted steel wire bonded to the six mandibular anterior teeth by the indirect method.
**Egli et al. [10]** **(2017)** ***AJODO***	Compare direct and indirect bonded mandibular-fixed retainers and study post-treatment changes after two years.	Randomized controlled trial (RCT)	64 consecutive patients were included in a two-arm RCT, according to an “indirect bonding group” versus a “direct bonding group”.	Of the 60 patients, five presented unexpected complications (all in the direct bonding group). **Prevalence:** 17%. **Observed movements:** Lingual crown inclination of 33. For one patient, the movement was considered clinically severe. **Apparition delay:** Two years. **Retainer:** 0.0215 inch, stainless steel, multistrand wire. Two bonding methods (direct and indirect) were employed.
**Jacobs et al. [19]** **(2017)** ***Head & Face Medicine***	Analyze the efficacy and accuracy of a completely customized lingual appliance regarding the correction of the torque of a single tooth.	Case series	Three patients who completed orthodontic treatment. Patients had a torque problem on one tooth with gingival recession.	The patients had unwanted movements. **Arch involved:** Mandible. **Observed movements:** Exaggerated torque on one tooth associated with gingival recession. **Retainer:** A bonded retainer without further details. For two patients, the wire was partially debonded. **Treatment:** Orthodontic retreatment with a completely customized lingual appliance associated with a reduction in the gingival recession.
**Roussarie et al. [24]** **(2018)** ***Revue*** ***d’Orthopédie*** ***Dento-Faciale***	Propose a mechanical theory to explain the apparition of Wire Syndrome.	Case series	115 cases. Patients’ documentation came from Dr. Roussarie’s office and from colleagues.	**Etiological hypotheses:** The bonded retention wire is “active” due to errors during bonding, during rebonding (repair), or due to an interposition of a hard foreign object; or, in presence of a fracture at the wire/bonding interface and when a force is applied, tooth can move/rotate around the wire. **Recommendation:** Wires should be passive and accurately rebonded if necessary. Retainer should be performed with the utmost care. Strengthening the wire/bonding interface (avoiding wire contamination before bonding, using a metal primer after degreasing the wire, sanding the wire surface to be bonded) should take place. In the case of Wire Syndrome (WS), the wire has to be removed, and a period of monitoring is recommended to achieve spontaneous repositioning. Patients should also be aware of the risk of unwanted movement associated with the presence of a fixed retainer.
**Beitlitum et al. [20]** **(2020)** ***International Journal of Environmental Research and Public Health***	Explore the benefits of a combined periodontic–orthodontic approach to resolve Miller class III gingival recession in post-orthodontic patients.	Prospective study	15 patients presented unexpected movements, despite the presence of a bonded retainer, associated with class III gingival recession were divided into two different groups.	**Two groups:** (1) Bonded lingual retainer removal prior to periodontal surgery plus removable retainer at night three months after surgery; (2) periodontal surgery only (without retainer removal). **Arch involved:** Mandible. **Retainer:** All patients had a lingual bonded retainer without further details. **Results:** For group (1), the improvement in the average recession depth was significantly greater (4.0 ± 0.83 mm; improvement: 87.2%) compared with group (2), who showed an improvement of 43.8% (1.88 ± 1.29 mm) (*p* = 0.008). Retainer removal prior to the surgery was beneficial in correcting Miller class III recessions.
**Kim et al. [21]** **(2020)** ***APOS Trends in Orthodontics***	Describe the types, causes, and recommendations for preventing/managing complications associated with bonded lingual retainers.	Case series	Nine patients who presented an intact fixed maxilla/mandibular retainer (no failure; no fracture).	Patients had unexpected tooth movements and gingival problems. **Arches involved:** Mandible and maxilla. **Retainer:** Maxillary or mandibular 0.0175 inch, multistrand wire bonded from canine to canine with a Duralay resin transfer method and a removable retainer (both arches) for night wearing. **Observed movements:** Several types of complications were described: Change in the transverse position, angulation, or torque of the crown, gingival recession, and non-specific complications such as space openings, misalignment, and appearance of black triangle.
**Klaus et al. [22]** **(2020)** ***BMC Oral Health***	Analyze the prevalence of undesirable tooth movement despite an intact fixed bonded retainer and identify possible predisposing factors.	Retrospective cohort study	Patients had completed previous orthodontic treatment and had a bonded canine-to-canine retainer. Patients with a removable retainer were excluded.	Of the 163 patients, 44 patients had adverse movements. **Prevalence:** 27%. **Arches involved:** Maxilla and mandible. **Retainer:** 0.018 inch, six-stranded coaxial wire. **Movement observed:** Maxillary retainers (20.9%) were more concerned than mandibular retainers (14%). Median amount of tooth movement: 0–0.66 mm with a large interindividual variation of up to 2.58 mm. **Risk factors:** These risk factors, associated with the occurrence of adverse movements, were dysfunction or parafunction (*p* = 0.049) and lacked inter-incisal contact (*p* < 0.01). No significant differences were found for the mandibular plane angle before treatment, amount of incisor proclination, expansion of the inter-canine distance, and overjet reduction during treatment.
**Knaup et al. [23]** **(2021)** ***Journal of Orofacial Orthopedics***	Measure tooth movement after retainer removal in cases of misalignment associated with a bonded retainer.	Case series (pilot study)	Patients completed orthodontic treatment, presented a fixed lingual retainer in the upper/lower jaw, and presented visible overcrowding. The existing retainers were removed to discontinue the present forces.	23 teeth were analyzed (12 upper teeth: 10 incisors, two canines; 11 lower teeth: 7 incisors, 4 canines). **Arches concerned**: Maxillary and mandibular. **Retainer:** Flexible, round spiral wire. **Observed movements:** Several types of movements were described and also measured. Misaligned teeth bonded to fixed retainers demonstrated movement when those retainers were debonded. These observations also highlight the impression that retainers might be able to provoke active force, which could be responsible for iatrogenic tooth movements.
**Singh et al. [5]** **(2021)** ***AJODO***	Describe a serious complication (canine completely avulsed) with a mandible bonded retainer	Case report	The patient (26 years old) completed orthodontic treatment with four premolar extractions 10 years earlier. The patient had received a mandibular bonded wire from canine to canine and removable retainers on both jaws. The removable retainers were prescribed the first year.	The patient had a severe complication that required the avulsion of the right mandibular canine. **Involved arch:** Mandible. **Observed movements:** 43 had torqued 70° labially, the apex was short and totally exposed. 42 presented lingual root torque. 32 was localized labially, and the apex was nearly exposed. An anterior and right lateral open bite was present. Generalized root resorptions from 20% to 40% were observed on the panoramic radiograph. The patient did not report any significant pain. **Retainer:** Supposition: Multistrand, twisted, fractured between 42 and 43. **Apparition delay:** 10 years. **Treatment:** Avulsion 43; retainer removal; nonsurgical periodontal therapy; six-month latency period. No further orthodontic intervention was advised because of the periodontal health and the presence of root resorptions.

**Table 4:** Wire Syndrome (WS) movement families. N.B.: The terms “X effect©” and “twist effect©” were introduced by Kucera et al. [11,16].

Movement Families Associated with WS
**X effect** Torque differential between two adjacent incisors
**Twist effect** Opposite inclination between the two contralateral canines
**Excessive crown and/or root torque** Incisors or canines, which presents an exaggerated modification of the torque
**Non-specific complications** Diastema openings, differences in incisor heights, etc.
**Associated movements** “X effect” AND Torque modification; “X effect” AND “twist effect” Or any other possible combination

**Table 5:** Etiological hypotheses of Wire Syndrome (WS).

**Practitioner-Related Etiologies [1,2,4,5,10,11,12,16,18,20,21,22]**
- Insufficient passivity
- Wire iatrogenic deformation during bonding
**Wire-related etiologies [1,2,4,5,10,11,12,14,16,18,19,20,21,22,24]**
- Wire deformation (chewing force or hard foods, traumatic application after dental floss, other harmful habits, or by parafunctions such as onychophagia)
- Modification or instability of the mechanical properties of wires: Wire fatigue, wire activation, and “despiralization” of the wire strands
- Adhesive failure at the adhesive/wire interface with the application of an external force
- Undetected wire debonded
- Fracture of the wire (remaining bonded to one or more teeth)
**Patient-related etiologies [18,19]**
- Physiological changes

**Table 6:** Recommendations to avoid Wire Syndrome (WS).

Recommendations to Bond the Retainer
- Careful manufacture of a passive wire on a dental model (precise adaptation, avoid any stress) [4,19,21]
- Indirect bonding method is indicated to avoid wire deformation by finger or instrument pressure [4,21]
- Bonding canines and central incisors only to improve patient sensitivity in the case of debonding [4]
- Strengthen the wire/adhesive liaison (avoid wire contamination before bonding, using a metal primer after degreasing the wire and sanding the wire surface to be bonded [24])
- Prescribe a removable retainer, in addition to the fixed retainer, for nighttime wear [4,5,18,21]
**Recommendations for follow-up after retainer placement**
- Regular check-ups [4,5,13,15,19]
- Early detection with systematic search for all signs of WS during retainer visits [1,2,4,5,11,13,14,15,21]
- Educate and inform patients about WS [1,2,11,13,15,21,24]
- Educate and inform all dental practitioners (general practitioners, periodontists, and orthodontists) about WS [2,3,5,13,21]

## Data Availability

The data underlying this article are available in the article.
